# Job satisfaction declines before retirement in Germany

**DOI:** 10.1007/s10433-024-00830-0

**Published:** 2024-11-11

**Authors:** Georg Henning, Graciela Muniz-Terrera, Andreas Stenling, Martin Hyde

**Affiliations:** 1 German Centre of Gerontology, Manfred-von-Richthofen-Straße 2, 12101 Berlin, Germany; 2 Ohio University Heritage College of Osteopathic Medicine, Ohio University, Athens, USA; 3 Centre for Clinical Brain Sciences and Centre for Dementia Prevention, University of Edinburgh, Edinburgh, UK; 4 Department of Psychology, University of Umeå, Umeå, Sweden; 5 Department of Sport Science and Physical Education, University of Agder, Kristiansand, Norway; 6 School of Management, University of Leicester, Leicester, UK

**Keywords:** Retirement, Job satisfaction, Latent growth curve modeling, Time-to-retirement approach

## Abstract

Job satisfaction has been found to increase with age. However, we still have a very limited understanding of how job satisfaction changes as people approach retirement. This is important as the years before retirement present specific challenges for older workers. We employed a time-to-retirement approach to investigate (i) mean levels of change in job satisfaction in the decade before retirement, and (ii) social inequalities and interindividual differences in change in pre-retirement job satisfaction. Data were drawn from the German Socioeconomic Panel Study (*n* = 2595). Latent growth curve modeling revealed that job satisfaction declined slightly as people approached retirement, with steeper declines in the very last years before retirement. However, the mean-level decline was very small. Education, age, health, region, marital status, and historical time, but not gender or caregiving mattered for level and change in job satisfaction before retirement. The findings demonstrate the importance of taking a time-to-retirement approach when examining experiences of older workers.

**Supplementary Information:**

The online version contains supplementary material available at 10.1007/s10433-024-00830-0.

## Introduction

Job satisfaction is important both for the individual, as it is associated with better mental and physical health (Satuf et al. 2018), and for the employer, as it is a strong predictor of higher productivity and lower turnover intentions (Woznyj et al. 2022). Older workers are often more satisfied at work than younger workers, and longitudinal studies show increases in job satisfaction across the work lifespan (Orth et al. 2012). However, the very last years of work, before workers retire, have rarely been studied. Workers face new challenges in this life phase, not least because retirement is approaching and workers start to prepare and likely disengage from their work life to some degree (Damman et al. 2013). Management and colleagues may also start to prepare for losing the worker and the experience of work for the older worker may therefore change in the very last years of work (Henning et al. 2023b). In the current study, we examine job satisfaction in the last ten work years from a process-based perspective, using time-to-retirement as the time metric, instead of chronological age. This approach can help us move from studying chronological age differences or changes with chronological age to one at the core interests of lifespan researchers—modeling typical and atypical development across the lifespan; here, in the last years of work.

### Age and job satisfaction

As older workers make up a large share of the workforce and are expected to work longer and retire later, their job satisfaction is a key issue (Hertel and Zacher 2018). It is important to investigate when and under which circumstances job satisfaction changes in late work life, to support fulfilling working lives. Cross-sectional studies have shown that older workers tend to report higher job satisfaction than younger workers (for a meta-analysis, see Ng and Feldman 2010). Although relatively few longitudinal studies on change in job satisfaction have been conducted, the evidence supports the assumption that job satisfaction increases with age (Dobrow Riza et al. 2018; McAdams et al. 2012; Orth et al. 2012).

There are two main explanations for positive age effects on job satisfaction. First, there are selection effects. Throughout working life people change jobs and companies and they are more likely to stay in those environments that make them happy (Garthe and Hasselhorn 2021; Ng and Feldman 2010). Therefore, older workers have simply had more time to find a satisfying job or form them in line with their needs, which can be seen as a type of job crafting (Wong and Tetrick 2017). Second, older workers may be better able to find satisfaction in work. Older workers show improved emotion regulation strategies to deal with daily stressors as work (Doerwald et al. 2016), and older workers tend to focus on emotion regulation rather than knowledge acquisition and are thus more likely to engage in satisfying activities at work (Kooij et al. 2011).

### Job satisfaction in the last work years—a process-based perspective

Earlier studies on age differences implied that job satisfaction may continuously increase until retirement, but they typically did not consider retirement timing. This raises a number of methodological issues. For example, if more satisfied workers work longer (Mein et al. 2000; Zacher and Rudolph 2017), cross-sectional age differences will be exaggerated because of selection of dissatisfied older (but not younger) workers into retirement. In longitudinal studies, the estimation of missing data in models of change is usually based on the assumption that dropout is not related to changes in the dependent variable (i.e., data are missing at random). If this is not true (e.g., if older adults are more likely to retire and thus “drop out” of the study once declines in job satisfaction occur), change parameters will be distorted. In the present paper, we use a process-based approach to focus on the development of job satisfaction in the years before retirement. This perspective implies that the proximity to retirement is a better explanation of late work-life developments than chronological age. Instead of investigating aging-related development, we study retirement-related development, focusing on the last years of employment as an important stage in the work life.

In the last years of work, workers face particular tasks and challenges. On the one hand, work is still a large part of one’s life, requires time and mental resources, and is important for well-being and identity; on the other hand, retirement is nearing, which requires psychological and organizational preparation and some degree of disengagement from work (Damman et al. 2013; Henning et al. 2023b). Individuals must balance investments in their current work situation with investments in their private life sphere and in planning and preparation for life in retirement (Shane and Heckhausen 2019). How well individuals manage this may have long-term consequences for well-being and health in older age.

Although there is little research which focuses on changes in job satisfaction in the last years of work, a recent study found declines in autonomous work motivation (Henning et al. 2023a, b), which is conceptually very similar to job satisfaction, in the years preceding retirement. There are also several reasons to assume declines in job satisfaction directly before retirement. First, older workers may partly disengage from work tasks and rather focus on the preparation for retirement in the last years before retirement (Damman et al. 2013; Henning et al. 2023b; Shane and Heckhausen 2019). Such disengagement may include reducing one’s work hours, stop keeping up with new developments at work, and handing over responsibilities to younger employees (Damman et al. 2013). Although such activities may help to prepare for retirement, they may affect satisfaction at work negatively, as daily activities at the workplace may become less interesting and fulfilling. Apart from anticipative disengagement processes, other internal and external factors may also lead to changes in job satisfaction in the last years of work. As workers age, perceived work ability declines (Ilmarinen et al. 1997), which may make it harder to fulfil one’s job tasks, which in turn may be related to declines in satisfaction. Again, such changes may often be blurred in studies focusing on chronological age if those with stronger declines in physical health retire earlier. Furthermore, well-designed and fit-for-purpose workplace training is assumed to lead to higher job satisfaction (Huang 2019); however, several studies have shown that older adults are seldom offered, but are also less interested in training opportunities, compared to younger workers (Hyde and Phillipson 2014; Karpinska et al. 2015). Changes in the workplace, for example the use of new technologies, may be perceived as problematic by older workers, if they do not receive adequate training, and decrease satisfaction. In addition, given that management and colleagues are likely aware that the older worker is approaching their retirement age, they may also integrate them less in their team and give them less important work tasks (Henning et al. 2023b). Finally, there may be selection effects: Workers may choose to retire once they experience declines in job satisfaction, because declines in job satisfaction are often followed by increased retirement intentions (Zacher and Rudolph 2017). If many people choose to retire when they become less satisfied, pre-retirement declines in satisfaction are likely.

In the current study, we do not aim to test which of these pathways is the dominant reason for potential declines in job satisfaction, but we aim to describe the resulting average development of job satisfaction at the end of one’s work life. Taken together, we assume:

#### H1

Job satisfaction will show mean-level declines in the 10 years before retirement.

### Interindividual differences

The main aim of this study is to understand mean-level changes in job satisfaction in the last years before retirement. However, older workers likely differ in their trajectories and levels of job satisfaction. Predictors are likely to be found among the individual job conditions, but the individual and macro-level socioeconomic context, specific health conditions, and one’s family life are likely to influence the course of job satisfaction as well. To be able to do justice to the predictors both in terms of theoretical reasoning and discussion of results, we limit ourselves to three main sociodemographic factors which are likely to be central dimensions of social inequalities or interindividual differences: gender, education, and age. However, we also test for the stability of their effects by controlling for marital status, self-rated health, caregiver status, region, and the year of retirement.

Both work-life courses (Engels et al. 2019; Nutz and Lersch 2021) and retirement transitions (Hofäcker et al. 2016) are strongly structured by gender in Germany: Women and men differ in work content, work hours, salary, retirement timing, and retirement characteristics. Women in midlife are also more likely to care for sick family members than men (Klaus and Vogel 2019,) and this may lead to work-family conflicts (Marks et al. 2002). The last years before retirement may also differ because transitions into family care often lead to (permanent) work exits for women (Ehrlich et al. 2020). Nevertheless, results of studies on job satisfaction in Germany are mixed (Bonsang and van Soest 2012), but usually they show small or no significant gender differences (Bönte and Krabel 2014; Hauret and Williams 2017). This has been explained by different frames of reference and expectations (Bonsang and van Soest 2012). However, gender differences in job satisfaction in the last work years before retirement are seldom studied.

Education is also a central determinant of work-life trajectories (Möhring et al. 2021) and retirement characteristics (Hess 2018) in Germany. Older workers with lower education have a higher risk of poor work quality (Hasselhorn et al. 2020), and often have to retire earlier than those with higher education (Rohrbacher and Hasselhorn 2022). However, little is known about differences in job satisfaction between those with higher and lower education among older German workers. Given the lack of results and clear theoretical arguments for or against specific gender and educational differences, we do not state specific hypotheses.

The third potential predictor of job satisfaction trajectories that we focus on in the current study is age at retirement. There are two reasons to assume that an older age at retirement is associated with different pre-retirement levels and trajectories of job satisfaction. First, if there is a typical age-related trajectory of job satisfaction, and it increases with age and these increases should become stronger later in life (McAdams et al. 2012). Hence, those retiring later would have a higher level and should have experienced greater increases in satisfaction. Second, if people with lower levels and declines in job satisfaction plan to retire earlier (Garthe and Hasselhorn 2023; Zacher and Rudolph 2017) and really leave the job at younger ages, this would only leave those with higher levels and more positive trajectories in job satisfaction in work at older ages. Consequently, our hypotheses are:

#### H2a

Compared to workers retiring at a younger age, an older age at retirement is related to higher job satisfaction before retirement.

#### H2b

Compared to workers retiring at a younger age, an older age at retirement is related to smaller decreases in job satisfaction before retirement.

## Method

### Sample

Our analyses were based on longitudinal data of the German Socioeconomic Panel (SOEP, Goebel et al. 2019), an ongoing representative German household panel study, with annual measurements from 1984. In the current analysis, we used data up to 2019, to avoid possible effects of the Covid-19 pandemic. We included everyone for whom a retirement year was identifiable (i.e., 1985–2019), which is, who were working in one wave and retired at the next. We defined retirement as receiving pension benefits without working for pay. This definition in line with earlier studies on the subject in Germany (Pinquart and Schindler 2007). We included only those who retired from work (instead of, for example, retiring after unemployment) to have a comparable transition point for every participant. We further included only those who were eligible to receive pensions (i.e., those aged 60 or older in the year of retirement) but allowed also people who were only 59 as they may have become 60 after the interview. We excluded very late transitions (i.e., later than age 67) because contracts end automatically when reaching the full retirement age, which is currently being raised from 65 to 67 but was below 66 for the cohorts included and those retiring later are likely a very selective group. A more detailed description of our selection procedure can be found in Appendix A. We focused on the last ten years of employment before receiving an old age pension. This decision was somewhat arbitrary, as there is little research so far informing us when job satisfaction starts to decline in late work life. We chose this time span to capture both development in late work life in general as well as the very last years before workers retire. We further believe that including ten years constitutes a good balance between choosing too many years, as estimates of change may be biased by the few people who were in the SOEP for decades before retirement, and too few years, as estimates in the last years may only capture the pre-retirement process and not typical developments in late working life.

The use of full information maximum likelihood (FIML) allowed us to include all available information in the analyses and handle missing data within the estimated models. To reduce bias as much as possible, we included those who reported job satisfaction at least once. Therefore, individuals contributed between one and ten data points to the study. Our selection criteria resulted in a sample of *n* = 2595 contributing with data on job satisfaction at least once.

### Measures

#### Job satisfaction

Participants were asked “How satisfied are you with your job?” on an 11-point Likert scale ranging from 0 (completely dissatisfied) to 10 (completely satisfied). Such single items of job satisfaction usually show high reliability and validity (Matthews et al. 2022).

#### Education

Workers were divided in three educational groups (low, middle, and high), based on the International Standard Classification of Education (ISCED). We included two dummy variables for low and high education, compared to middle education, in the analyses.

#### Gender

Gender was included as a dichotomous variable (0 = female, 1 = male).

#### Age

We included age at the year of retirement (60 to 66). We centered age at 60, so the variable had the values 0 to 6.

#### Control variables

We included marital status (1 = married, 0 = not married), self-rated health, caregiving, and region (former West = 0, former East = 1), as well as retirement year as control variables to predict job satisfaction. Information on all covariates except age at retirement was taken from the last interview before retirement. Self-rated health was assessed on a 5-point scale from bad to very good. Health was grand-mean centered in our analyses, which means that level and slopes can be interpreted as the estimated values for those reporting self-rated health at the sample mean. We included information on caregiving from a time use questionnaire. If participants reported spending some time during weekdays or weekends “Looking after and treatment of people requiring care,” they were coded as providing care. The variable was dummy coded (1 = caregiver, 0 = not caregiver). Retirement year was re-centered (− 34 = 1985 − 0 = 2019), so the intercept of the models including this variable can be interpreted for those retiring in 2019. Table 1 shows descriptive statistics of the study variables.

**Table 1:** Descriptive statistics

	*M* (*SD*)/*n (%)*
Age at retirement (*n* = 2595)	62.54 (2.08)
Gender (*n* = 2595)	Female = 1,064 (41.00%)
	Male = 1,531 (59.00%)
West/East German (*n* = 2595)	West = 1,940 (75.55%)
	East = 628 (24.45%)
Education (*n* = 2554)	Low = 352 (13.78%)
	Middle = 1,332 (65.94%)
	High = 870 (34.06%)
Marital status (*n* = 2595)	Married = 2,019 (77.80%) Not married = 576 (22.20%)
Self-rated health (*n* = 2.129)	3.16 (0.89)
Job satisfaction 1 year before retirement	6.90 (2.15)
Job satisfaction 2 years before retirement	7.02 (2.05)
Job satisfaction 3 years before retirement	6.99 (2.09)
Job satisfaction 4 years before retirement	7.13 (2.04)
Job satisfaction 5 years before retirement	7.11 (2.00)
Job satisfaction 6 years before retirement	7.20 (1.90)
Job satisfaction 7 years before retirement	7.10 (1.96)
Job satisfaction 8 years before retirement	7.09 (2.03)
Job satisfaction 9 years before retirement	7.11 (2.01)
Job satisfaction 10 years before retirement	7.08 (1.99)

*N* = 2595

### Analysis

To study how job satisfaction changes before retirement, we used latent growth curve modeling (Meredith and Tisak 1990; Ram and Grimm 2007) in MPlus version 8.4 (Muthén and Muthén 2020). Latent growth curve models include an intercept, often depicting the baseline level, a linear slope to capture linear change, and (often) a slope to incorporate nonlinear change (e.g., a quadratic slope). As we were interested in changes in the last years before retirement, we followed the literature on time-to-death models in gerontology (Muniz-Terrera et al. 2013) to compute time-to-retirement models on change in job satisfaction. Based on the individual retirement year, we re-structured the data on job satisfaction from a wave-metric to a time-to-retirement metric counting backwards from retirement. Individuals reported their job satisfaction on average for 6.76 years (*SD* = 3.28) in the last ten years before retirement. We used a model with a random intercept and random slopes.^1^ In contrast to usual latent growth curves, in our model, the intercept was set at 1 year before retirement. This means that the slopes are not to be interpreted as in growth curves that describe linear change. Instead, the linear slope can be interpreted as the tangent (the rate of decline/growth) at the last assessment before retirement. The quadratic slope illustrates the change in rate of change, which is the general form of development.

As a first step, we fitted an unconditional model without any predictors. If the estimated development showed a significant decline in the work years before retirement, this would be support for H1. To investigate interindividual differences (i.e., hypotheses H2a and H2b), we predicted the intercept and slopes by gender, education, and age in separate models as well as in a model with mutual adjustments, which also included control variables (education, gender, region, marital status, caregiver status, and self-rated health).

We inspected the global model fit based on the comparative fit index (CFI), the root mean square error of approximation (RMSEA), and the standardized root mean residual (SRMR). A CFI of 0.90 or higher, as well as a SRMR and RMSEA of 0.08 or lower is seen as acceptable fit to the data (Marsh 2007). The robust full information maximum likelihood estimator (MLR) was used to account for missing data (Enders 2010). The alpha level for all analyses was set to 0.05. For transparency and to allow replication, we provide a Stata syntax to generate our study sample, as well as Mplus input files to run all models https://osf.io/a78tf/.

## Results

### Change in job satisfaction

**Fig. 1:**
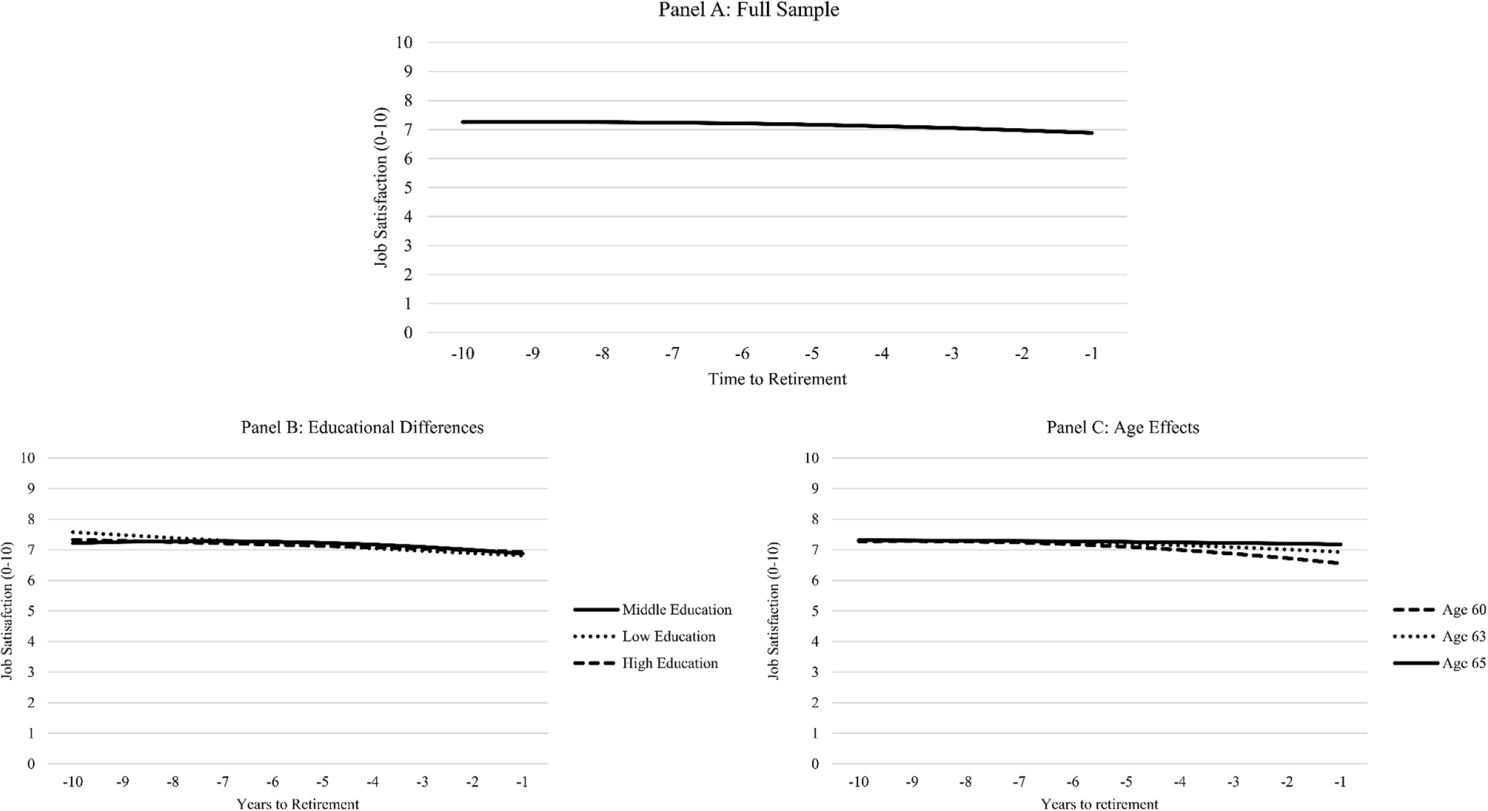
Job satisfaction before retirement

The unconditional model had a very good fit (CFI = 0.987, SRMR = 0.034, RMSEA = 0.021, 90% CI [0.015; 0.027]) and showed a significant negative linear slope (*M* = − 0.10, 95%CI[− 0.13; − 0.06], *p* < 0.001) and a small but significant negative quadratic slope (*M* = − 0.01, 95%CI[− 0.01; − 0.002], *p* = 0.001). As can be seen in Fig. 1, Panel A, it seems like job satisfaction was rather stable at the beginning of the last decade at work, until later on, when, in line with H1, job satisfaction declined when people got closer to retirement and there were distinct declines in the last years of one’s work life. However, as can be seen in Table 1, the changes were rather small, given that mean levels of job satisfaction were between 6.90 (at the last wave) and 7.20 (six years before retirement) in the years before retirement, which translates to standardized differences of only up to *d* = 0.15. Intercept and slopes all showed significant variances, which means that people differed in their pre-retirement level of job satisfaction and the rate of change before retirement.^2^ The positive intercept–slope correlation (*r* = 0.35, *p* < 0.001) shows that people with higher job satisfaction also showed more positive trajectories of job satisfaction leading up to retirement.^3^

**Table 2:** Results from fully adjusted latent growth model

	Level			Linear slope			Quadratic slope		
	B(SE)	95%CI		B (SE)	95%CI		B(SE)	95%CI	
		LL	UL		LL	UL		LL	UL
Intercept	6.16 (0.15)***	5.87	6.45	− 0.11 (0.06)	− 0.22	0.01	− 0.01 (0.01)	− 0.02	0.01
Gender (1 = Women)	− 0.16 (0.09)	− 0.33	0.01	− 0.04 (0.04)	− 0.11	0.02	0.01 (0.00)	− 0.01	0.00
Education (reference: middle)									
High	0.01 (0.09)	− 0.16	0.19	0.05 (0.04)	− 0.02	0.12	0.01 (0.00)*	0.00	0.02
Low	− 0.07 (0.13)	− 0.32	0.18	0.10 (0.05)	− 0.002	0.20	0.01 (0.01)*	0.00	0.02
Age (0 = 60 − 6 = 66)	0.14 (0.02)***	0.09	0.18	0.02 (0.01)	− 0.001	0.04	0.00 (0.00)	0.00	0.00
East Germany	− 0.36 (0.10)**	− 0.56	− 0.15	− 0.05 (0.04)	− 0.14	0.03	− 0.01 (0.01)*	− 0.02	− 0.001
Married	0.29 (0.10)**	0.08	0.49	0.05 (0.04)	− 0.03	0.13	0.01 (0.05)	− 0.004	0.01
Self− rated health	0.68 (0.05)***	0.58	0.78	0.07 (0.02)***	0.03	0.11	0.01 (0.00)*	0.00	0.01
Caregiving	0.02 (0.17)	− 0.31	0.34	0.01 (0.07)	− 0.12	0.14	0.00 (0.01)	− 0.01	0.02
Retirement year (− 34 = 1985 − 0 = 2019)	− 0.02 (0.01)***	− 0.03	− 0.01	0.01 (0.00)**	0.00	0.01	0.00 (0.00)	0.00	0.00

*p < 0.05 **p < 0.01 ***p < 0.001 *n* = 2,595. Model fit: CFI = 0.987, SRMR = 0.028, RMSEA = 0.016; 90%CI[0.012; 0.020]

### Individual differences and social inequalities

We ran separate models to investigate gender, educational and age differences, without mutual adjustment by including those variables as predictors of intercept and slopes. We did not observe any statistically significant gender differences in level (*B* = 0.06, 95%CI[− 0.11; 0.23], *p* = 0.486), linear (*B* = − 0.05, 95%CI[− 0.11; 0.02], *p* = 0.169), or quadratic slope (*B* = − 0.01, 95%CI[− 0.01; 0.00], *p* = 0.210).

We did not observe statistically significant effects of low or high education, contrast to medium education, on the pre-retirement level of job satisfaction (low education: *B* = − 0.07, 95%CI[− 0.31;0.19], *p* = 0.610; high education: *B* = 0.06, 95%CI[− 0.13;0.24], *p* = 0.554), however, those with higher education showed less decrease before retirement both in terms of the linear (*B* = 0.08, 95%CI[0.01; 0.14], *p* = 0.033) and the quadratic slope, which means that the form of change was flatter for this group (*B* = 0.01, 95%CI[0.001; 0.02], *p* = 0.023). Those with lower education showed a flatter decline before retirement as well, as shown by the statistically significant and positive effect on the quadratic slope (*B* = 0.01, 95%CI[0.00; 0.02]; *p* = 0.048). In Figure 1, Panel B illustrates the differences.^4^

In a model with age as a predictor, we found that those retiring at higher ages showed a higher job satisfaction before retirement (*B* = 0.12, 95%CI[0.09; 0.16], *p* < 0.001) as well as more positive changes (linear slope: *B* = 0.03, 95%CI[0.02; 0.05], *p* < 0.001, quadratic slope: *B* = 0.002, 95%CI[0.00; 0.004], *p* = 0.049) before retirement, in line with hypotheses 2a and 2b. This is illustrated in Fig. 1, Panel C – job satisfaction trajectories for those with a retirement at age 65 (i.e., the full retirement age) was mainly stable.

Finally, we included all variables and covariates in one model; coefficients can be found in Table 2.^5^ The positive effects of higher education on level of job satisfaction and the quadratic slope of job satisfaction remained statistically significant, but not on the linear slope. A lower education was still negatively associated with the quadratic slope. A higher age remained a statistically significant predictor of pre-retirement job satisfaction, but not of change. Of the covariates, those from West Germany, those who were married, those with better health, and those retiring earlier in historical time showed better pre-retirement job satisfaction. Those with better health and those retiring later in historical time showed more positive pre-retirement trajectories and a flatter trajectory. West German retirees showed flatter trajectories as well.

## Discussion

In the present study, we investigated trajectories in job satisfaction before retirement. Previous studies have often reported higher job satisfaction among older adults and increases with age (Ng and Feldman 2010), but it remained unclear if such findings can be generalized to the end of one’s work life. In the current study, we expected declines in job satisfaction in the last ten years before retirement.

### How does job satisfaction change before retirement?

In the German SOEP, job satisfaction remained relatively stable in late work life, but there were mean-level declines in the very last years before retirement. This is in line with H1 and an earlier study on change in work motivation before retirement (Henning et al. 2023b). Whereas older workers may be more satisfied than younger workers on average (Ng and Feldmann 2010), this does not mean that job satisfaction increases linearly across the work lifespan. Earlier longitudinal studies showing increases in work satisfaction across the work lifespan (e.g., Orth et al. 2012) may partly suffer from selection effects. At least in Germany, the last years of work may rather be characterized by some declines. It should be noted, though, that the size of these declines was rather small in our sample.

Our analyses cannot show the mechanisms behind the pre-retirement decline. However, one possible reason may be that people decide to retire when job satisfaction declines, although German workers typically plan when to retire rather early in their life and seem to stick to these plans (Engstler 2019). Alternatively, a decreasing quality of the individual work experience in the pre-retirement years may be responsible. Such changes may be the consequence of physical and cognitive declines or of age discrimination at work, but it may also be the consequence of a disengagement process to prepare for retirement. Damman et al. (2013) showed that people proactively change their behavior at work toward retirement and this may be associated with a decreasing quality of work. More studies are needed to shed light on changes in subjective and objective work conditions in the last years of work and their relation with retirement and work motivation (Stiller et al. 2021), as well as possible disengagement processes (Damman et al. 2013).

### Interindividual differences

We were interested in testing if men and women, and those with high, middle, and low education differed in their job satisfaction before retirement. We found no gender differences, which is in line with earlier studies (Bonsang and van Soest 2012; Bönte and Krabel 2014) and may be explained by different expectations or comparison groups. In contrast, we found significant differences in job satisfaction and change in job satisfaction between educational groups, showing advantages for the high education group, who may benefit from more job autonomy, a higher social status, and better health, compared to those with middle education. Furthermore, higher educated older workers are likely to have more financial autonomy to choose when to retire, leaving only those who are still satisfied with their job in the work force. We found no disadvantages of low education, compared to those with middle education, in terms of pre-retirement job satisfaction or declines before retirement. However, it is important to note that we only included workers who retired directly from work in our study. Many workers, especially with lower education, do not retire directly but experience a period of unemployment or disability pension before taking out retirement pensions (Engstler and Romeu Gordo 2017). If these groups were considered, larger gender and educational differences may be found.

We further investigated if the trajectories differed between workers retiring at younger or older ages. Those who retired at older ages showed higher levels of satisfaction before retirement and less negative trajectories before, as we expected. This is in line with findings of positive age effects on job satisfaction (McAdams et al. 2012). Scholars have argued that adaptive self-regulation strategies can explain these effects (Ng and Feldman 2010). Alternatively, age-specific selection effects may be an explanation: As noted before, declines in job satisfaction may lead people to think about retirement (Zacher and Rudolph 2017). At older ages, when workers are eligible to receive full or only slightly reduced pensions, they have the opportunity to react accordingly and retire, leaving only those who are rather satisfied in the workforce. Declines in job satisfaction at younger ages, however, most likely have to be much more drastic for workers to retire even without sufficient income.

Among the control variables, self-rated health and historical time were related to change in job satisfaction. Individuals who felt healthier also showed less declines in job satisfaction before retirement—individuals with poor self-reported health probably feel less capable of keeping up with their work. This result highlights the need for occupational health management and for adjusting the workplace to the needs of workers with health problems, regardless of age. However, please note that we did not have a measure of disease load or physical functioning in the dataset, but only a single-item assessing self-rated health. Although such items constitute valid measures of physical and mental health (Mavaddat et al. 2011; Singh-Manoux et al. 2006), psychological constructs and well-being seem to play a part in self-evaluations of health as well, especially when people get older (Huisman and Deeg 2010; Lang et al. 2016; Layes et al. 2012; Spuling et al. 2015), and thus the effects of self-rated health on job satisfaction may to some degree be reflective of psychological traits and states instead of actual physical or mental health.

Concerning historical time, trajectories among those retiring later in historical time were more positive.^6^ This may be caused by historical changes in the work environments (Hülür et al. 2019), societal views on aging (Beyer et al. 2017), or cohort effects, for example in physical or cognitive functioning (Gerstorf et al. 2020). Recent research has shown that retirement adjustment can vary, depending on the historical time of the transition (Henning et al. 2023a), therefore future studies may investigate more in detail how the experience of the last years of work life have changed with historical time. Nevertheless, those retiring later in historical time also retired at lower levels of job satisfaction. This may relate to the increasingly restricted choice to select early retirement (Hess 2016, 2018), so dissatisfied workers remained employed. However, please note that most pension reforms mainly affected the opportunity to retire from unemployment or disability pension (Börsch-Supan et al. 2024) and the effects of more recent pension reforms like the gradually raised retirement age are most likely not yet visible in our dataset. Future studies may apply quasi-experimental approaches to investigate how job satisfaction changed after specific pension reforms.

In addition to timing of retirement, healthier, married, and West German individuals showed higher levels of job satisfaction before retirement. The advantage of married individuals in terms of job satisfaction has been found before (Saner and Eyüpoğlu 2013). The disadvantage of East Germans may have to do with different experiences on the labor market and problems after the unification (Jilke 2016). In more recent years, West and East German workers seem to show comparable job satisfaction (Brenke 2015). More research is needed to identify more specific factors that contribute to interindividual differences in job satisfaction in the last years of work, and if those factors differ from those in other work-life stages.

### Strengths and limitations

To our knowledge, this is the first study to investigate change in job satisfaction from a time-to-retirement perspective and increases our knowledge on psychological development in late work life. The use of a representative sample, the investigation of nonlinear within-person change, as well as the use of more meaningful time metrics (time-to-retirement) have often been missing in previous studies in the field (Bohlmann et al. 2018). Further strengths of our study include the large sample size and the use of a large time span (ten years).

One major limitation of our study is that we only describe, but not explain, changes before retirement. The literature offers different reasons for such effects, including disengagement processes, changes at the workplace, or selection effects as retirement may often happen once declines in job satisfaction have started, which may be investigated in future studies. Furthermore, we used only single-item measures of job satisfaction. More nuanced measures may reveal more complexity, and better handle potential measurement error. As job satisfaction is an individual evaluation, our results do not inform about changes in objective work conditions. In particular those with less resources and more constraints in the labor market may have learned to accept more negative work environments and recalibrated their standards accordingly.

Our results are only representative for the German context and for those who work until retirement, not for other countries or workers with indirect transitions. However, indirect transitions (e.g., from unemployment) have become more frequent in the last years and it is therefore important that future studies investigate how job satisfaction develops before people get unemployed or start to receive disability pensions before retirement.

## Conclusion

Our study showed that job satisfaction declined toward the end of one’s work life in Germany, although only to a small degree. Our findings further highlight that the individual agency among older workers to leave a negative work environment is restricted by the available financial resources and that the development of the pension system, restricting opportunities for earlier retirement, may have an impact on job satisfaction and health of older workers. Given the increases in the general retirement age over the next years in Germany and in many other countries, as well as the demographic change (Hertel and Zacher 2018; van Dalen et al. 2015), it is an important task for future studies to identify who is able to stay comparably satisfied at work and who may suffer in the last years of a prolonged working life. Our results should be an encouragement to study the last years of work, and under which circumstances older workers can maintain job satisfaction and motivation while planning and preparing for retirement at the same time.

## Supplementary Information

Below is the link to the electronic supplementary material.

Supplementary file1 (DOCX 99 kb)

## Footnotes

1 Details on the model selection can be found in the appendix.

2 All parameters of this model can be found in the appendix, Table A2.

3 In Appendix C, we report a sensitivity analysis for people who reported retirement between age 18 and 59 (based on self-reported retirement status). They showed even more pronounced declines in job satisfaction before retirement.

4 Please note that although according to the figure, the lower educated would have had the highest job satisfaction ten years before retirement, we assume that this is due to the complicated modeling approach and the limited data on these earlier waves. A linear regression showed no effects of low education on job satisfaction ten years before retirement.

5 Effects of the covariates without mutual adjustment can be found in the appendix.

6 The effects of historical time were very similar when no other covariates or only age was controlled for.

## References

[CR1] Beyer A-K, Wurm S, Wolff JK (2017) Älter werden–Gewinn oder Verlust? Individuelle Altersbilder und Altersdiskriminierung. In Altern im Wandel, Springer. pp 329–343

[CR2] Bohlmann C, Rudolph CW, Zacher H (2018) Methodological recommendations to move research on work and aging forward. Work, Aging Retire 4(3):225–237. 10.1093/workar/wax023

[CR3] Bonsang E, van Soest A (2012) Satisfaction with job and income among older individuals across European countries. Soc Indic Res 105(2):227–254. 10.1007/s11205-011-9879-5 10.1007/s11205-011-9886-6 PMC3228937 22207781

[CR4] Bönte W, Krabel S (2014) You can’t always get what you want: gender differences in job satisfaction of university graduates. Appl Econ 46(21):2477–2487. 10.1080/00036846.2014.899677

[CR5] Börsch-Supan AH, Rausch J, Salerno L (2024) Pension reforms and inequality in Germany: micro-modelling (No. w32796). National Bureau of Economic Research. 10.3386/w32796

[CR6] Brenke K (2015) Die große Mehrzahl der Beschäftigten in Deutschland ist mit ihrer Arbeit zufrieden. Diw Wochenbericht 82(32/33):715–722

[CR7] Damman M, Henkens K, Kalmijn M (2013) Late-career work disengagement: the role of proximity to retirement and career experiences. J Gerontol B Psychol Sci Soc Sci 68(3):455–463. 10.1093/geronb/gbt001 23407785 10.1093/geronb/gbt001

[CR8] Dobrow Riza S, Ganzach Y, Liu Y (2018) Time and job satisfaction: a longitudinal study of the differential roles of age and tenure. J Manag 44(7):2558–2579

[CR9] Doerwald F, Scheibe S, Zacher H, Van Yperen NW (2016) Emotional competencies across adulthood: State of knowledge and implications for the work context. Work, Aging Retire 2(2):159–216. 10.1093/workar/waw013

[CR10] Ehrlich U, Möhring K, Drobnič S (2020) What comes after caring? The impact of family care on women’s employment. J Fam Issues 41(9):1387–1419

[CR11] Enders CK (2010) Applied missing data analysis. Guilford press, New York

[CR12] Engels M, Weyers S, Moebus S, Jöckel K-H, Erbel R, Pesch B, Behrens T, Dragano N, Wahrendorf M (2019) Gendered work-family trajectories and depression at older age. Aging Ment Health 23(11):1478–1486. 10.1080/13607863.2018.1501665 30621439 10.1080/13607863.2018.1501665

[CR13] Engstler H (2019) Wie erfolgreich sind ältere Arbeitskräfte in der zeitlichen Umsetzung ihrer Ausstiegspläne? Z Gerontol Geriatr 52(1):14–24. 10.1007/s00391-018-1451-3 30267263 10.1007/s00391-018-1451-3

[CR14] Engstler H, Gordo LR (2017) Der Übergang in den Ruhestand: Alter, Pfade und Ausstiegspläne. In: Mahne K, Wolff JK, Simonson J, Tesch-Römer C (eds) Altern im Wandel. Springer Fachmedien Wiesbaden, Wiesbaden, pp 65–80. 10.1007/978-3-658-12502-8_4

[CR15] Garthe N, Hasselhorn HM (2021) Leaving and staying with the employer—Changes in work, health, and work ability among older workers. Int Arch Occup Environ Health 94:85–93 32893311 10.1007/s00420-020-01563-0 PMC7826300

[CR16] Garthe N, Hasselhorn H (2023) Wollen und können ältere Beschäftigte länger erwerbstätig bleiben, wenn sich ihre Arbeit verbessert? Zentralblatt für Arbeitsmedizin, Arbeitsschutz und Ergonomie,. 10.1007/s40664-022-00490-w

[CR17] Gerstorf D, Hülür G, Drewelies J, Willis SL, Schaie KW, Ram N (2020) Adult development and aging in historical context. Am Psychol 75(4):525–539. 10.1037/amp0000596 32378947 10.1037/amp0000596

[CR18] Goebel J, Grabka MM, Liebig S, Kroh M, Richter D, Schröder C, Schupp J (2019) The German socio-economic panel (SOEP). Jahrbücher für Nationalökonomie und Statistik, 239(2):345–360. 10.1515/jbnst-2018-0022

[CR19] Hasselhorn HM, Stiller M, du Prel J-B, Ebener M (2020) Work profiles of older employees in Germany-results from the lidA-cohort study. BMC Public Health 20(1):1452. 10.1186/s12889-020-09542-3 32977775 10.1186/s12889-020-09542-3 PMC7519553

[CR20] Hauret L, Williams DR (2017) Cross-national analysis of gender differences in job satisfaction. Ind Relat: A J Econ Soc 56(2):203–235

[CR21] Henning G, Baumann I, Huxhold O (2023a) Historical and cross-country differences in life satisfaction across retirement in Germany and Switzerland from 2000 to 2019. J Gerontol: Ser B 78(8):1365–1374. 10.1093/geronb/gbad066 10.1093/geronb/gbad066 PMC10394993 37293925

[CR22] Henning G, Stenling A, Tafvelin S, Ebener M, Lindwall M (2023) Levels and change in autonomous and controlled work motivation in older workers–the role of proximity to retirement and sense of community at work. J Occupat Organiz Psychol 96(1):33–55. 10.1111/joop.12406

[CR23] Hertel G, Zacher H (2018) Managing the aging workforce. In: Ones D, Anderson N, Sinangil H, Viswesvaran C (eds) The SAGE handbook of industrial, work and organizational psychology. SAGE Publications Ltd, London, pp 396–425. 10.4135/9781473914964.n19

[CR24] Hess M (2016) Germany: A successful reversal of early retirement? In: Hofäcker D, Hess M, König S (eds) Delaying retirement. Springer, pp 147–169

[CR25] Hess M (2018) Expected and preferred retirement age in Germany. Z Gerontol Geriatr 51(1):98–104 27125820 10.1007/s00391-016-1053-x

[CR26] Hofäcker D, Schröder H, Li Y, Flynn M (2016) Trends and determinants of work-retirement transitions under changing institutional conditions: Germany, England and Japan compared. J Soc Policy 45(1):39–64

[CR27] Huang W-R (2019) Job training satisfaction, job satisfaction, and job performance. Career Dev Job Satisfaction, 25.

[CR28] Huisman M, Deeg DJ (2010) A commentary on Marja Jylhä’s “What is self-rated health and why does it predict mortality? Towards a unified conceptual model” (69:3, 2009, 307-316). Soc Sci Med 70(5):652–657. 10.1016/j.socscimed.2009.11.003 19942333 10.1016/j.socscimed.2009.11.003

[CR29] Hülür G, Ram N, Willis SL, Schaie KW, Gerstorf D (2019) Cohort differences in cognitive aging: the role of perceived work environment. Psychol Aging 34(8):1040–1054. 10.1037/pag0000355 31804111 10.1037/pag0000355

[CR30] Hyde M, Phillipson C (2014) How can lifelong learning, including continuous training within the labour market, be enabled and who will pay for this? Looking forward to 2025 and 2040 how might this evolve. Future of an ageing population: evidence review.

[CR31] Ilmarinen J, Tuomi K, Klockars M (1997) Changes in the work ability of active employees over an 11-year period. Scand J Work Environ Health 23:49–57 9247995

[CR32] Jilke S (2016) Job satisfaction and regime change: Evidence from a natural experiment. Int Public Manag J 19(3):370–396. 10.1080/10967494.2015.1043168

[CR33] Karpinska K, Henkens K, Schippers J, Wang M (2015) Training opportunities for older workers in the Netherlands: a vignette study. Res Soc Stratification Mobility 41:105–114

[CR34] Klaus D, Vogel C (2019) Unbezahlte Sorgetätigkeiten von Frauen und Männern im Verlauf der zweiten Lebenshälfte. In: Vogel C, Wettstein M, Tesch-Römer C (eds) Frauen und Männer in der zweiten Lebenshälfte: Älterwerden im sozialen Wandel. Springer Fachmedien Wiesbaden, Wiesbaden, pp 91–112. 10.1007/978-3-658-25079-9_6

[CR35] Kooij DTAM, De Lange A, Jansen P, Kanfer R, Dikkers J (2011) Age and work-related motives: results of a meta-analysis. J Organ Behav 32:197–225. 10.1002/job.665

[CR36] Lang FR, Gerstorf D, Weiss D, Wagner GG (2016) On differentiating adaptation from disposition concepts: the case of age-associated dynamics of life satisfaction. J Individ Differ 37(3):206–210. 10.1027/1614-0001/a000205

[CR37] Layes A, Asada Y, Kepart G (2012) Whiners and deniers - what does self-rated health measure? Soc Sci Med 75(1):1–9. 10.1016/j.socscimed.2011.10.030 22265085 10.1016/j.socscimed.2011.10.030

[CR38] Marks NF, Lambert JD, Choi H (2002) Transitions to caregiving, gender, and psychological well-being: a prospective US national study. J Marriage Fam 64(3):657–667

[CR39] Marsh HW (2007) Application of confirmatory factor analysis and structural equation modeling in sport and exercise psychology. In: Tenenbaum G, Eklund RC (eds) Handbook of sport psychology. Wiley, pp 774–798. 10.1002/9781118270011.ch35

[CR40] Matthews RA, Pineault L, Hong Y-H (2022) Normalizing the use of single-item measures: validation of the single-item compendium for organizational psychology. J Bus Psychol, pp 1–36

[CR41] Mavaddat N, Kinmonth AL, Sanderson S, Surtees P, Bingham S, Khaw KT (2011) What determines self-rated health (SRH)? A cross-sectional study of SF-36 health domains in the EPIC-Norfolk Cohort. J Epidemiol Commun Health 65(9):800–806. 10.1136/jech.2009.090845 10.1136/jech.2009.090845 20551149

[CR42] McAdams KK, Lucas RE, Donnellan MB (2012) The role of domain satisfaction in explaining the paradoxical association between life satisfaction and age. Soc Indic Res 109(2):295–303. 10.1007/s11205-011-9903-9

[CR43] Mein G, Martikainen P, Stansfeld SA, Brunner EJ, Fuhrer R, Marmot MG (2000) Predictors of early retirement in British civil servants. Age Ageing 29(6):529–536. 10.1093/ageing/29.6.529 11191246 10.1093/ageing/29.6.529

[CR44] Meredith W, Tisak J (1990) Latent curve analysis. Psychometrika 55:107–122

[CR45] Möhring K, Weiland A, Reifenscheid M, Naumann E, Wenz A, Rettig T, Krieger U, Fikel M, Cornesse C, Blom AG (2021) Inequality in employment trajectories and their socio-economic consequences during the early phase of the COVID-19 pandemic in Germany.

[CR46] Muniz-Terrera G, van den Hout A, Piccinin AM, Matthews FE, Hofer SM (2013) Investigating terminal decline: results from a UK population-based study of aging. Psychol Aging 28(2):377 23276221 10.1037/a0031000 PMC3692590

[CR47] Muthén L, Muthén B (2020) Mplus. The comprehensive modelling program for applied researchers: user’s guide, 5.

[CR48] Ng TWH, Feldman DC (2010) The relationship of age with job attitudes: a meta analysis. Personnel Psychol 63(3):677–718. 10.1111/j.1744-6570.2010.01184.x

[CR49] Nutz T, Lersch PM (2021) Gendered employment trajectories and individual wealth at older ages in Eastern and Western Germany. Adv Life Course Res 47:100374. 10.1016/j.alcr.2020.100374 36695142 10.1016/j.alcr.2020.100374

[CR50] Orth U, Robins RW, Widaman KF (2012) Life-span development of self-esteem and its effects on important life outcomes. J Pers Soc Psychol 102(6):1271 21942279 10.1037/a0025558

[CR51] Pinquart M, Schindler I (2007) Changes of life satisfaction in the transition to retirement: a latent-class approach. Psychol Aging 22(3):442–455. 10.1037/0882-7974.22.3.442 17874946 10.1037/0882-7974.22.3.442

[CR52] Ram N, Grimm K (2007) Using simple and complex growth models to articulate developmental change: Matching theory to method. Int J Behav Dev 31(4):303–316. 10.1177/0165025407077751

[CR53] Rohrbacher M, Hasselhorn HM (2022) Social inequalities in early exit from employment in Germany: a causal mediation analysis on the role of work, health, and work ability. Scandinavian J Work Environ Health 48(7):569–578. 10.5271/sjweh.4043 10.5271/sjweh.4043 PMC10539108 35708627

[CR54] Saner T, Eyüpoğlu ŞZ (2013) The gender-marital status job satisfaction relationship of academics. Procedia Soc Behav Sci 106:2817–2821. 10.1016/j.sbspro.2013.12.324

[CR55] Satuf C, Monteiro S, Pereira H, Esgalhado G, Marina Afonso R, Loureiro M (2018) The protective effect of job satisfaction in health, happiness, well-being and self-esteem. Int J Occup Saf Ergon 24(2):181–189 27560543 10.1080/10803548.2016.1216365

[CR56] Shane J, Heckhausen J (2019) Motivational theory of lifespan development. In: Work across the lifespan. Elsevier. pp 111–134

[CR57] Singh-Manoux A, Martikainen P, Ferrie JE, Zins M, Marmot MG, Goldberg M (2006) What does self rated health measure? Results from the British Whitehall II and French Gazel cohort studies’. J Epidemiol Community Health 60(4):364–372. 10.1136/jech.2005.039883 16537356 10.1136/jech.2005.039883 PMC2566175

[CR58] Spuling SM, Wurm S, Tesch-Römer C, Huxhold O (2015) Changing predictors of self-rated health: disentangling age and cohort effects. Psychol Aging 30(2):462 25961881 10.1037/a0039111

[CR59] Stiller M, Garthe N, Hasselhorn HM (2021) Job quality trajectories among baby-boomers in Germany and their consequences for the motivation to work – results from the lidA cohort study. Ageing Soc. 10.1017/S0144686X21001343

[CR60] van Dalen HP, Henkens K, Wang M (2015) Recharging or retiring older workers? Uncovering the age-based strategies of European employers. Gerontologist 55(5):814–824. 10.1093/geront/gnu048 24898558 10.1093/geront/gnu048

[CR61] Wong CM, Tetrick LE (2017) Job crafting: older workers’ mechanism for maintaining person-job fit. Front Psychol. 10.3389/fpsyg.2017.01548 10.3389/fpsyg.2017.01548 PMC5596060 28943859

[CR62] Woznyj HM, Banks GC, Whelpley CE, Batchelor JH, Bosco FA (2022) Job attitudes: a meta-analytic review and an agenda for future research. J Organ Behav 43(5):946–964

[CR63] Zacher H, Rudolph CW (2017) Change in job satisfaction negatively predicts change in retirement intentions. Work, Aging Retire 3(3):284–297. 10.1093/workar/wax009

