# Supplementary material for: Job satisfaction declines before retirement in Germany
Source: Eur J Ageing. 2024 Nov 11;21(1):33. doi: 10.1007/s10433-024-00830-0 (PMC11554980; doi:10.1007/s10433-024-00830-0)
Supplement: Supplementary file 1 — Supplementary file1 (DOCX 99 kb) [file 10433_2024_830_MOESM1_ESM.docx]

**Appendix**

**A Definition of retirement and sample selection procedure**

We restricted the sample to those whose reported age at retirement was feasible, given the mandatory retirement age in the German pension system. Germany has been called an “early exit” country (Ebener et al., 2019) as most retirees do not work up to the general retirement age, which was 65 for most of the study period. Many retirees use early retirement options, even if this means accepting pension cuts. Depending on the birth cohort, type of work, and other life circumstances, early retirement was possible from age 60 in Germany and is currently possible from age 63. To reduce complexity, we included everyone who was aged between 60 and 66 in the year of retirement, without considering the eligibility rules at the specific year and exact months of birth or retirement. Many workers also stopped working before they were eligible for pensions, for example because they lost their jobs or got sick. However, in the current paper, we focus on those individuals retiring directly from work, as this is the normative transition type, and because declines in work satisfaction for those with earlier work exits may largely be driven by anticipating disability or unemployment.

Only including those with a direct work to retirement transition is a more conservative test of our hypotheses, as people who stopped working already before official retirement may show stronger declines in job satisfaction in the last work years and indirect retirement is becoming more frequent (Engstler & Romeu Gordo, 2017). In Germany, workers have limited opportunities to choose their retirement age, in contrast to other countries where retirement and retirement age are optional and of the individual’s choice (Wetzel et al., 2016).

The individual retirement year was defined as the year when someone switched from receiving salary and no pension to receiving pension. We included only those who did not receive salary in the year after retirement to exclude working retirees. We included only those retiring from 1985 onwards, to have data on participants from work life at least once before the retirement year, and only those who made only one retirement transition. This led to a sample of *n* = 4,056.

We further excluded those who we excluded those who were younger than 59 (*n* = 723) or older than 67 (*n* = 48) at the year of retirement. Those younger than 59 could not receive pension for legal reasons and were thus likely to receive disability pensions, and those older than 67 were a very selective group. The resulting sample size was *n* = 3,258. We next excluded those who received salary without working because they used a special offer (called “Altersteilzeit”) that allowed them to retire earlier while still receiving part-time salary until reaching the retirement age (*n* = 341). Finally, we excluded those who never reported job satisfaction over the course of the study (*n* = 349). The final study sample size was *n* = 2,595.

**B Model selection**

To select the right model for the type of development in our study, we started with a model with only a (fixed) intercept, and added components (random intercept, fixed linear slope, random linear slope, fixed quadratic slope, random quadratic slope) if model fit (χ²) improved significantly. In addition to χ² differences, we further investigated change in the Akaike information criterion (AIC) and Bayesian information criterion (BIC). A model with lower AIC and BIC indicates a better model fit compared to a model with higher AIC and BIC. Model fit and test statistics can be found in Table A1.

The χ² - model fit improved significantly with increased model complexity; AIC and BIC also showed consistently decreased values. However, please note that after introduction of a fixed quadratic slope, BIC decreased only to a small degree (ΔBIC = 0.58) and ΔBIC values of less than 2 have been called “not worth more than a bare mention” (Fabozzi et al., 2014). We selected the most complex model with a random intercept, a random linear, and a random quadratic slope.

*Table A1.* Model fit and test statistics of different growth curves

|  | AIC | BIC | *χ²* | Satorra-Bentler Scaled *χ²* test |
| --- | --- | --- | --- | --- |
| Fixed intercept | 71196.13 | 71260.60 | 4770.24, *df* = 54 | *-* |
| Random intercept | 64956.69 | 65027.02 | 529.58, *df* = 53 | *χ²(*1*) =* 1,036.28, *p <* .001 |
| Random intercept, fixed linear slope | 64848.25 | 64924.45 | 452.84, *df* = 52 | *χ²(*1*) =* 56.88*, p <* .001 |
| Random intercept, random linear slope | 64454.15 | 64542.07 | 161.00, *df* = 50 | *χ²(*2*) =* 294.02*, p <* .001 |
| Random intercept, random linear slope, fixed quadratic slope | 64447.71 | 64541.49 | 154.42, *df* = 49 | *χ²(*2*) =* 6.60, *p =* .037 |
| Random intercept, random linear slope, random quadratic slope | 64377.42 | 64488.78 | 99.06, *df* = 46 | *χ²(*3*) =* 52.42*, p <* .001 |

Finally, we tested for homoscedasticity (i.e., equal residual variances over time) and found that such a model did not differ significantly in model fit (*χ²(*9*) =* 11.74, *p =* .228), so we continued with it in the following analyses. Table A2 shows parameters of the final model.

*Table A2.* Parameter estimates of the unconditional latent growth curve.

|  | Intercept | Linear Slope | Quadratic Slope |
| --- | --- | --- | --- |
| Mean (*SE*) | 6.89 (0.04)*** | -0.10 (0.02)*** | -0.01 (0.00)** |
| Variance | 2.90 (0.14)*** | 0.14 (0.03)*** | 0.001 (0.00)*** |
| Covariances | | | |
| Intercept- Linear Slope | 0.22 (0.04)*** | | |
| Intercept – Quadratic Slope | 0.00 (0.01) | | |
| Linear Slope – Quadratic Slope | 0.01 (0.00)*** | | |
| Correlations | | | |
| Intercept- Linear Slope | 0.34 (0.05)*** | | |
| Intercept – Quadratic Slope | 0.07 (0.07) | | |
| Linear Slope – Quadratic Slope | 0.87 (0.03)*** | | |

* *p* < .05 *** *p* < .001; *n* = 2,595

**C Effects of covariates without mutual adjustment**

East Germany retirees showed a lower job satisfaction before retirement (*B* = -0.40, 95%CI[-0.61;-0.19], *p* < .001), but no significant differences were found concerning change (linear slope: *B* = -0.04, 95%CI[-0.12;0.04], *p* = .319, quadratic slope: *B* = -0.01, 95%CI[-0.02;0.00], *p* = .054).

Better health was associated with a higher level of job satisfaction (*B* = -0.73, 95%CI[0.63;0.83], *p* < .001) as well as weaker decreases before retirement (*B* = 0.08, 95%CI[0.04;0.12], *p* < .001) and a flatter trajectory (*B* = 0.004, 95%CI[0.00;0.01], *p* = .041).

Those who were married showed a higher job satisfaction before retirement (*B* = 0.27, 95%CI[0.06;0.47], *p* < .001), but no significant differences were found concerning change (linear slope: *B* = 0.03, 95%CI[-0.05;0.11], *p* = .046, quadratic slope: *B* = 0.00, 95%CI[-0.01;0.01], *p* = .560).

Those who retired later in historical time showed a lower job satisfaction before retirement (*B* = -0.01, 95%CI[-0.02;-0.002], *p* = .016) and less linear declines (*B* = 0.01, 95%CI[0.00;0.01], *p* < .001), but no different form of change (i.e., quadratic change, (*B* = 0.00, 95%CI[0.00;0.00], *p* = .089).

Care did have no effect on level (*B* = -0.04, 95%CI[-0.39;0.31], *p* = .808), linear slope (*B* = 0.05, 95%CI[-0.09;0.18], *p* = .493) or quadratic slope (*B* = 0.00, 95%CI[-0.01;0.02], *p* = .734).

**D Sensitivity Analysis**

As mentioned in the method part of the paper, many workers retire before they are eligible for pensions. We therefore repeated our latent growth curve model with a different subsample. For this analysis, we used a different variable in the data set which is based on self-reported work status. We choose the retirement year as the year when people changed from receiving salary and not reporting retirement to reporting being retired. Again, we only included those who did not receive any salary in the following year, and only those who reported some data on job satisfaction. This led to a sample of *n* = 668 participants retiring between 18 and 58 (*M* = 51.35, *SD* = 7.03). In this sample, repeating the growth curve model led to a model with a lower job satisfaction at the last wave before retirement (*B* = 6.16, *SE* = 0.09, *p* < .001), a steeper linear decline (*B* = -0.22, *SE* = 0.06, *p* < .001) and a similar quadratic slope decline (*M* = -0.01, SE = 0.00, *p* = .004). This is illustrated in Figure A1.

**Figure A1**. Trajectories of retirees aged 18-58 at retirement**
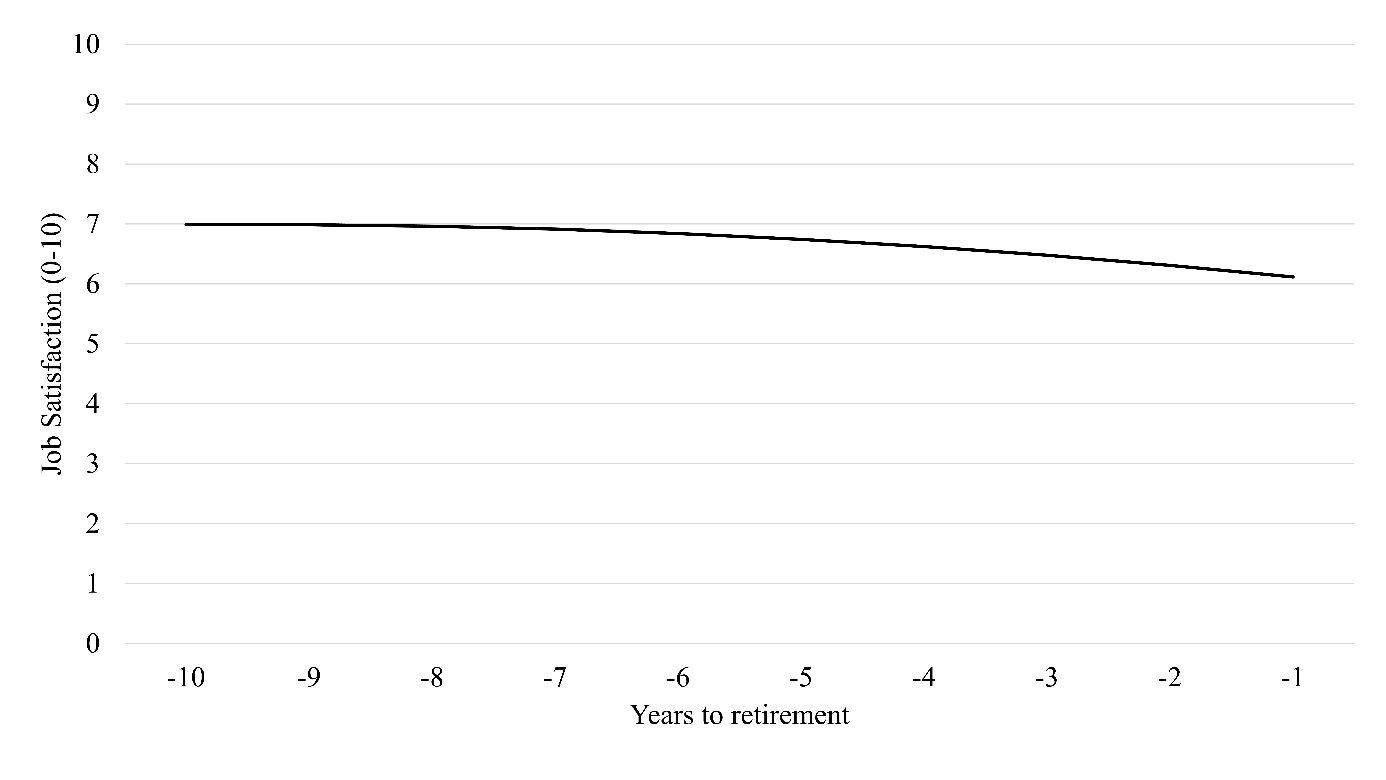
**

**References:**

Fabozzi, F. J., Focardi, S. M., Rachev, S. T., & Arshanapalli, B. G. (2014). *The basics of financial econometrics: Tools, concepts, and asset management applications.* John Wiley & Sons.

Ebener, M., Rings, A., & Hasselhorn, H. (2019). When does a social norm of “early exit” have impact on desired and planned retirement age? Results from the lidA cohort study. *Das Gesundheitswesen*, *81*(08/09), 1A-2.

Engstler, H., & Romeu Gordo, L. (2017). Der Übergang in den Ruhestand: Alter, Pfade und Ausstiegspläne. In K. Mahne, J. K. Wolff, J. Simonson, & C. Tesch-Römer (Eds.), *Altern im Wandel: Zwei Jahrzehnte Deutscher Alterssurvey (DEAS)* (pp. 65-80). Springer Fachmedien Wiesbaden. <https://doi.org/10.1007/978-3-658-12502-8_4>

Wetzel, M., Huxhold, O., & Tesch-Römer, C. (2016). Transition into retirement affects life satisfaction: Short-and long-term development depends on last labor market status and education. *Social Indicators Research*, *125*(3), 991-1009. <https://doi.org/10.1007/s11205-015-0862-4>
